# Deacetyl­nomilin monohydrate

**DOI:** 10.1107/S1600536811035033

**Published:** 2011-09-03

**Authors:** Guo-Qiang Li, Yong-Shu Ye, Yi-Ting Yang, Hu-Jie Luo, Yao-Lan Li

**Affiliations:** a Guangdong Province Key Laboratory of Pharmacodynamic Constituents of ­Traditional Chinese Medicine and New Drugs Research, Institute of Traditional Chinese Medicine and Natural Products, College of Pharmacy, Jinan University, Guangzhou 510632, People’s Republic of China; b Infinitus (China) Company Ltd, Guangzhou 510665, People’s Republic of China

## Abstract

In the title compound (systematic name 1-hy­droxy-1,2-dihydro­obacunoic acid 3,4-lactone monohydrate), C_26_H_32_O_8_·H_2_O, the dihedral angles between the planes of the ester groups and the furan plane are 43.06 (12) and 56.06 (7)°, while that between the furan plane and the keto group is 58.50 (9)°. The *A*/*B*, *B*/*C* and *C*/*D* ring junctions are all *trans*-fused. Inter­molecular O—H⋯O hydrogen bonds between the hy­droxy and carbonyl groups and the water mol­ecule give rise to a three-dimensional structure.

## Related literature

For general background to the title compound, see: Dreyer (1965 ▶); Munehiro *et al.* (1989 ▶). For the absolute configuration of (−)-nomilin, see: Zhang *et al.* (2006 ▶). For details of ring conformations and puckering parameters, see: Cremer & Pople (1975 ▶); Boeyens (1978 ▶).
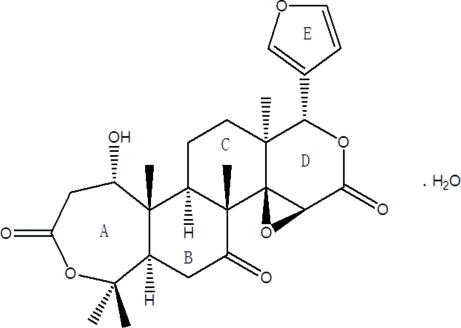


## Experimental

### 

#### Crystal data

C_26_H_32_O_8_·H_2_O
*M* *_r_* = 490.53
Orthorhombic, 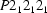

*a* = 10.6037 (2) Å
*b* = 13.6564 (3) Å
*c* = 16.2893 (4) Å
*V* = 2358.82 (9) Å^3^
*Z* = 4
Cu *K*α radiation
μ = 0.86 mm^−1^
*T* = 298 K
0.42 × 0.23 × 0.20 mm

#### Data collection

Oxford Diffraction Xcalibur Sapphire3 Gemini Ultra CCD diffractometer
Absorption correction: multi-scan (*CrysAlis PRO*; Agilent, 2011 ▶) *T*_min_ = 0.819, *T*_max_ = 1.000
5397 measured reflections
3378 independent reflections
3206 reflections with *I* > 2σ(*I*)
*R*_int_ = 0.019

#### Refinement

*R*[*F* ^2^ > 2σ(*F* ^2^)] = 0.032
*wR*(*F* ^2^) = 0.085
*S* = 1.03
3378 reflections
329 parameters
1 restraint
H atoms treated by a mixture of independent and constrained refinement
Δρ_max_ = 0.14 e Å^−3^
Δρ_min_ = −0.17 e Å^−3^
Absolute structure: Flack (1983 ▶), 1213 Friedel pairs
Flack parameter: −0.19 (1)

### 

Data collection: *CrysAlis PRO* (Agilent, 2011 ▶); cell refinement: *CrysAlis PRO*; data reduction: *CrysAlis PRO*; program(s) used to solve structure: *SHELXS97* (Sheldrick, 2008 ▶); program(s) used to refine structure: *SHELXL97* (Sheldrick, 2008 ▶); molecular graphics: *OLEX2* (Dolomanov *et al.*, 2009 ▶); software used to prepare material for publication: *OLEX2*.

## Supplementary Material

Crystal structure: contains datablock(s) I, global. DOI: 10.1107/S1600536811035033/zs2135sup1.cif

Structure factors: contains datablock(s) I. DOI: 10.1107/S1600536811035033/zs2135Isup2.hkl

Additional supplementary materials:  crystallographic information; 3D view; checkCIF report

## Figures and Tables

**Table 1:** Hydrogen-bond geometry (Å, °)

*D*—H⋯*A*	*D*—H	H⋯*A*	*D*⋯*A*	*D*—H⋯*A*
O3—H3⋯O9	0.82	2.04	2.817 (3)	158
O9—H9*A*⋯O6^i^	0.85 (1)	2.00 (1)	2.841 (3)	169 (4)
O9—H9*B*⋯O4^ii^	0.87 (5)	2.33 (5)	3.124 (3)	152 (4)

Symmetry codes: (i) 
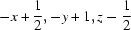
; (ii) 
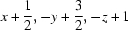
.

**Fig. 1.**
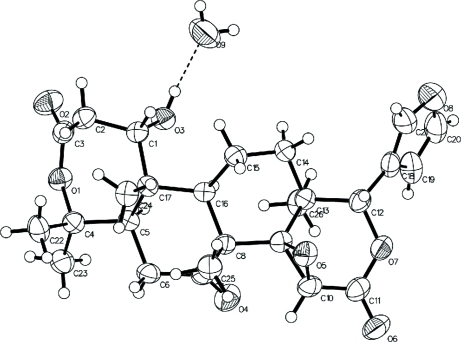
The molecular structure of the title compound showing 50% probability displacement ellipsoids and the atom-numbering scheme.

**Fig. 2.**
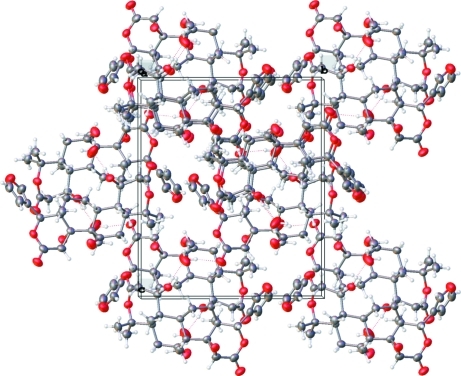
The packing in the unit cell viewed down the a axis.

## supplementary crystallographic information

### Comment

The title compound C_26_H_32_O_8_ . H_2_O (Fig. 1) is the monohydrate of deacetylnomilin (systematic name: 1-hydroxy-1,2-dihydroobacunoic acid 3,4 -lactone monohydrate), which was originally isolated from the seeds of the genera *Citrus* and *Poncirus* (Dreyer, 1965; Munehiro *et al.*, 1989). With the present compound, which was isolated from the traditional Chinese medicine Pericarpium Citri Reticulatae, the dihedral angles between the planes of the ester groups and the furan plane are 43.06 (12)° and 56.06 (7)°, while that between the furan plane and the keto group is 58.50 (9)°. The title compound is composed of five rings, one seven–membered, one five-membered and three six–membered. The seven–membered ring (*A*) adopts a chair conformation as does the six–membered ring (*B*), which has puckering parameters (Cremer & Pople, 1975; Boeyens, 1978) Q = 0.588 (2) Å, θ = 5.2 (2)°, φ = 239 (2)°. The rings *C* and *D* adopt skew–boat conformations with puckering paramaters Q = 0.775 (2) Å, θ = 98.35 (15)°, φ = 94.92 (15)° and Q = 0.512 (2) Å, θ = 110.4 (2)°, φ = 92.9 (2)°, respectively. The *A*/*B*, *B*/*C* and *C*/*D* ring junctions are all *trans*- fused. Intermolecular O—H···O hydrogen bonds (Table 1) involving the hydroxy and carbonyl groups and the water molecule give a three-dimensional structure (Fig. 2). The absolute configuration determined for the parent (-)-nomilin (Zhang *et al.*, 2006) was invoked, giving the assignments C1(*S*), C5(*R*), C8(*R*), C9(*R*), C10(*S*), C12(*S*), C13(*S*), C16(*R*), C17(*R*) for the 9 chiral centres in the molecule (using the numbering scheme employed in Fig. 1).

### Experimental

The title compound was isolated from the traditional Chinese medicine Pericarpium Citri Reticulatae, 500g of which was extracted with boiling water, then concentrated by rotary evaporator. The crude extract was subjected to silica gel column chromatography, eluted using a methanol/chloroform gradient. Further purification of the cloroform/methanol (92/8) fraction by silica gel column chromatography with EtOAc/cyclohexane (35/65) gave the title compound (4 mg). Crystals of the title compound were obtained after slow evaporation of a methanolic solution at room temperature.

### Refinement

The C-bound H atoms were positioned geometrically and were included in the refinement in the riding-model approximation, with C—H = 0.93 Å (alkenyl H), 0.96 Å (CH_3_), 0.97 Å (CH_2_), 0.98 Å (CH) and O—H = 0.82 Å and with *U*_iso_(H) = 1.2*U*_eq_(C) (alkenyl, methylene and methine) and = 1.5*U*_eq_[C(methyl) and O]. The absolute structure determined for (-)-nomilin (Zhang *et al.*, 2006) was invoked: the Flack parameter determined for the parent compound not being definitive [-0.19 (1) for 1213 Friedel pairs].

### Crystal data

**Table:**

C_26_H_32_O_8_·H_2_O	*D*_x_ = 1.381 Mg m^−^^3^
*M**_r_* = 490.53	Cu *K*α radiation, λ = 1.5418 Å
Orthorhombic, *P*2_1_2_1_2_1_	Cell parameters from 3366 reflections
*a* = 10.6037 (2) Å	θ = 3.2–62.6°
*b* = 13.6564 (3) Å	µ = 0.86 mm^−^^1^
*c* = 16.2893 (4) Å	*T* = 298 K
*V* = 2358.82 (9) Å^3^	Prism, colourless
*Z* = 4	0.42 × 0.23 × 0.20 mm
*F*(000) = 1048	

### Data collection

**Table:**

Oxford Diffraction Xcalibur Sapphire3 Gemini Ultra CCD diffractometer	3378 independent reflections
Radiation source: Enhance Ultra (Cu) X-ray Source	3206 reflections with *I* > 2σ(*I*)
mirror	*R*_int_ = 0.019
Detector resolution: 16.0288 pixels mm^-1^	θ_max_ = 62.7°, θ_min_ = 4.2°
ω scans	*h* = −12→11
Absorption correction: multi-scan (*CrysAlis PRO*; Agilent, 2011)	*k* = −15→9
*T*_min_ = 0.819, *T*_max_ = 1.000	*l* = −15→18
5397 measured reflections	

### Refinement

**Table:**

Refinement on *F*^2^	Secondary atom site location: difference Fourier map
Least-squares matrix: full	Hydrogen site location: inferred from neighbouring sites
*R*[*F*^2^ > 2σ(*F*^2^)] = 0.032	H atoms treated by a mixture of independent and constrained refinement
*wR*(*F*^2^) = 0.085	*w* = 1/[σ^2^(*F*_o_^2^) + (0.0494*P*)^2^ + 0.2836*P*] where *P* = (*F*_o_^2^ + 2*F*_c_^2^)/3
*S* = 1.03	(Δ/σ)_max_ < 0.001
3378 reflections	Δρ_max_ = 0.14 e Å^−^^3^
329 parameters	Δρ_min_ = −0.17 e Å^−^^3^
1 restraint	Absolute structure: Flack (1983), 1213 Friedel pairs
Primary atom site location: structure-invariant direct methods	Flack parameter: −0.19 (1)

### Special details

**Table:**

Geometry. All e.s.d.'s (except the e.s.d. in the dihedral angle between two l.s. planes) are estimated using the full covariance matrix. The cell e.s.d.'s are taken into account individually in the estimation of e.s.d.'s in distances, angles and torsion angles; correlations between e.s.d.'s in cell parameters are only used when they are defined by crystal symmetry. An approximate (isotropic) treatment of cell e.s.d.'s is used for estimating e.s.d.'s involving l.s. planes.
Refinement. Refinement of *F*^2^ against ALL reflections. The weighted *R*-factor *wR* and goodness of fit *S* are based on *F*^2^, conventional *R*-factors *R* are based on *F*, with *F* set to zero for negative *F*^2^. The threshold expression of *F*^2^ > σ(*F*^2^) is used only for calculating *R*-factors(gt) *etc*. and is not relevant to the choice of reflections for refinement. *R*-factors based on *F*^2^ are statistically about twice as large as those based on *F*, and *R*- factors based on ALL data will be even larger.

### Fractional atomic coordinates and isotropic or equivalent isotropic displacement parameters (Å^2^)

**Table:**

	*x*	*y*	*z*	*U*_iso_*/*U*_eq_	
O1	0.14223 (12)	1.05987 (10)	0.53350 (8)	0.0411 (3)	
O3	0.23637 (13)	0.84306 (10)	0.45658 (8)	0.0419 (3)	
H3	0.2592	0.8159	0.4142	0.063*	
O5	0.44209 (13)	0.58155 (10)	0.73498 (8)	0.0427 (3)	
O4	0.13428 (16)	0.74314 (12)	0.75935 (11)	0.0580 (4)	
O2	0.14192 (16)	1.07137 (13)	0.40087 (9)	0.0592 (4)	
O6	0.21183 (16)	0.44886 (12)	0.84070 (10)	0.0594 (4)	
O7	0.26007 (14)	0.42551 (10)	0.71158 (8)	0.0462 (4)	
O8	0.2620 (3)	0.29900 (14)	0.45914 (12)	0.0827 (6)	
C3	0.19938 (19)	1.04622 (14)	0.46131 (13)	0.0402 (5)	
C17	0.34443 (17)	0.88604 (13)	0.58418 (11)	0.0311 (4)	
C10	0.3221 (2)	0.57743 (16)	0.77603 (12)	0.0428 (5)	
H10	0.3120	0.6192	0.8245	0.051*	
C2	0.3284 (2)	1.00257 (14)	0.45659 (13)	0.0414 (4)	
H2A	0.3861	1.0439	0.4871	0.050*	
H2B	0.3555	1.0028	0.3997	0.050*	
C5	0.22296 (17)	0.92924 (13)	0.62505 (10)	0.0319 (4)	
H5	0.1527	0.8962	0.5974	0.038*	
C15	0.43123 (19)	0.70549 (14)	0.55659 (12)	0.0381 (4)	
H15A	0.4517	0.7327	0.5032	0.046*	
H15B	0.5087	0.6996	0.5879	0.046*	
C26	0.14800 (18)	0.58545 (14)	0.60780 (13)	0.0418 (5)	
H26A	0.1291	0.6541	0.6051	0.063*	
H26B	0.1028	0.5565	0.6527	0.063*	
H26C	0.1231	0.5545	0.5574	0.063*	
C11	0.2616 (2)	0.48032 (15)	0.77933 (13)	0.0451 (5)	
C16	0.33892 (17)	0.77336 (13)	0.60152 (11)	0.0307 (4)	
H16	0.2558	0.7535	0.5812	0.037*	
C8	0.33860 (18)	0.74107 (13)	0.69383 (11)	0.0331 (4)	
C6	0.2122 (2)	0.89767 (15)	0.71652 (12)	0.0422 (5)	
H6A	0.2792	0.9287	0.7476	0.051*	
H6B	0.1324	0.9208	0.7384	0.051*	
C14	0.3705 (2)	0.60464 (14)	0.54619 (12)	0.0403 (5)	
H14A	0.4364	0.5567	0.5367	0.048*	
H14B	0.3170	0.6058	0.4979	0.048*	
C1	0.33849 (18)	0.89800 (13)	0.48969 (11)	0.0341 (4)	
H1	0.4163	0.8699	0.4674	0.041*	
C7	0.2202 (2)	0.78844 (15)	0.72810 (11)	0.0387 (4)	
C19	0.1464 (2)	0.35218 (16)	0.56310 (17)	0.0573 (6)	
H19	0.0785	0.3626	0.5981	0.069*	
C24	0.47170 (17)	0.93032 (15)	0.61138 (13)	0.0405 (5)	
H24A	0.4921	0.9850	0.5768	0.061*	
H24B	0.5366	0.8816	0.6069	0.061*	
H24C	0.4656	0.9519	0.6673	0.061*	
C9	0.33382 (18)	0.62767 (14)	0.69640 (11)	0.0344 (4)	
C13	0.29043 (17)	0.57139 (14)	0.62084 (11)	0.0342 (4)	
C23	0.0774 (2)	1.06898 (17)	0.66732 (13)	0.0497 (5)	
H23A	0.0486	1.1325	0.6505	0.075*	
H23B	0.0991	1.0705	0.7245	0.075*	
H23C	0.0117	1.0218	0.6585	0.075*	
C4	0.19324 (19)	1.04057 (14)	0.61709 (12)	0.0374 (4)	
C25	0.4526 (2)	0.77543 (16)	0.74614 (14)	0.0465 (5)	
H25A	0.5296	0.7580	0.7188	0.070*	
H25B	0.4497	0.7444	0.7990	0.070*	
H25C	0.4491	0.8452	0.7530	0.070*	
C18	0.2691 (2)	0.39381 (14)	0.57069 (13)	0.0434 (5)	
C21	0.3345 (3)	0.35915 (16)	0.50605 (15)	0.0616 (7)	
H21	0.4183	0.3743	0.4949	0.074*	
C22	0.2986 (2)	1.11287 (15)	0.63681 (15)	0.0493 (5)	
H22A	0.3660	1.1049	0.5980	0.074*	
H22B	0.3296	1.1007	0.6912	0.074*	
H22C	0.2665	1.1785	0.6336	0.074*	
C20	0.1471 (3)	0.29599 (17)	0.49702 (18)	0.0644 (7)	
H20	0.0786	0.2594	0.4788	0.077*	
C12	0.31795 (19)	0.46163 (14)	0.63545 (12)	0.0392 (4)	
H12	0.4095	0.4529	0.6396	0.047*	
O9	0.3772 (3)	0.74718 (16)	0.33392 (16)	0.0972 (8)	
H9A	0.351 (4)	0.6885 (14)	0.329 (3)	0.146*	
H9B	0.431 (4)	0.752 (3)	0.293 (3)	0.129 (16)*	

### Atomic displacement parameters (Å^2^)

**Table:**

	*U*^11^	*U*^22^	*U*^33^	*U*^12^	*U*^13^	*U*^23^
O1	0.0374 (7)	0.0466 (7)	0.0392 (7)	0.0056 (6)	−0.0025 (6)	0.0043 (6)
O3	0.0418 (7)	0.0472 (7)	0.0365 (7)	−0.0089 (6)	−0.0064 (6)	−0.0033 (6)
O5	0.0352 (7)	0.0476 (8)	0.0453 (8)	0.0078 (6)	−0.0121 (6)	0.0058 (6)
O4	0.0504 (9)	0.0554 (9)	0.0682 (10)	0.0080 (8)	0.0211 (9)	0.0130 (8)
O2	0.0604 (10)	0.0724 (10)	0.0448 (8)	0.0074 (9)	−0.0090 (8)	0.0125 (8)
O6	0.0657 (10)	0.0636 (10)	0.0488 (9)	0.0012 (9)	0.0103 (8)	0.0182 (8)
O7	0.0538 (9)	0.0401 (7)	0.0448 (8)	−0.0002 (7)	−0.0012 (7)	0.0086 (6)
O8	0.1235 (18)	0.0628 (11)	0.0619 (11)	−0.0171 (12)	0.0025 (13)	−0.0107 (9)
C3	0.0456 (11)	0.0362 (10)	0.0387 (10)	−0.0033 (9)	−0.0035 (10)	0.0041 (9)
C17	0.0256 (9)	0.0340 (9)	0.0338 (10)	−0.0016 (8)	−0.0032 (8)	−0.0022 (8)
C10	0.0459 (11)	0.0495 (11)	0.0330 (9)	0.0060 (10)	−0.0050 (9)	0.0049 (9)
C2	0.0401 (10)	0.0445 (10)	0.0395 (10)	−0.0049 (9)	0.0026 (9)	0.0041 (9)
C5	0.0283 (8)	0.0353 (9)	0.0320 (9)	−0.0001 (8)	−0.0007 (8)	−0.0029 (8)
C15	0.0347 (10)	0.0404 (10)	0.0391 (11)	0.0046 (9)	0.0038 (9)	−0.0005 (8)
C26	0.0360 (10)	0.0377 (10)	0.0517 (12)	0.0021 (9)	−0.0112 (10)	0.0027 (9)
C11	0.0423 (11)	0.0496 (12)	0.0433 (11)	0.0088 (10)	−0.0030 (10)	0.0117 (10)
C16	0.0245 (8)	0.0372 (9)	0.0302 (9)	−0.0006 (8)	−0.0006 (8)	−0.0012 (8)
C8	0.0326 (9)	0.0368 (9)	0.0300 (9)	0.0011 (8)	−0.0035 (8)	0.0002 (8)
C6	0.0470 (11)	0.0459 (11)	0.0337 (10)	0.0104 (10)	0.0053 (9)	−0.0024 (9)
C14	0.0487 (11)	0.0353 (9)	0.0369 (10)	0.0055 (9)	0.0032 (9)	−0.0011 (9)
C1	0.0277 (9)	0.0394 (9)	0.0351 (10)	−0.0059 (8)	0.0017 (8)	−0.0001 (8)
C7	0.0415 (10)	0.0451 (10)	0.0295 (9)	0.0036 (10)	−0.0006 (9)	0.0033 (9)
C19	0.0556 (14)	0.0407 (11)	0.0755 (16)	−0.0016 (11)	−0.0116 (13)	−0.0010 (12)
C24	0.0288 (9)	0.0427 (10)	0.0501 (11)	−0.0054 (9)	−0.0061 (9)	−0.0001 (10)
C9	0.0279 (9)	0.0414 (10)	0.0337 (10)	0.0067 (9)	−0.0058 (8)	0.0039 (8)
C13	0.0317 (9)	0.0358 (9)	0.0351 (9)	0.0034 (8)	−0.0035 (8)	0.0017 (8)
C23	0.0533 (13)	0.0476 (11)	0.0481 (12)	0.0155 (11)	0.0057 (11)	−0.0003 (10)
C4	0.0370 (10)	0.0397 (10)	0.0356 (10)	0.0029 (8)	−0.0044 (9)	−0.0008 (8)
C25	0.0497 (12)	0.0471 (12)	0.0427 (11)	−0.0013 (10)	−0.0168 (10)	−0.0013 (10)
C18	0.0516 (12)	0.0306 (9)	0.0479 (11)	0.0008 (9)	−0.0073 (10)	0.0048 (9)
C21	0.0777 (17)	0.0480 (12)	0.0590 (14)	−0.0140 (13)	0.0107 (14)	−0.0097 (12)
C22	0.0537 (13)	0.0368 (10)	0.0574 (13)	−0.0023 (10)	−0.0100 (11)	−0.0068 (10)
C20	0.0799 (19)	0.0411 (11)	0.0721 (17)	−0.0100 (13)	−0.0256 (16)	0.0015 (12)
C12	0.0370 (10)	0.0391 (10)	0.0413 (10)	0.0040 (9)	−0.0010 (9)	0.0065 (9)
O9	0.130 (2)	0.0746 (14)	0.0870 (15)	−0.0275 (14)	0.0387 (15)	−0.0335 (12)

### Geometric parameters (Å, °)

**Table:**

O1—C3	1.336 (2)	C8—C7	1.519 (3)
O1—C4	1.489 (2)	C8—C9	1.550 (3)
O3—H3	0.8200	C8—C25	1.552 (3)
O3—C1	1.424 (2)	C6—H6A	0.9700
O5—C10	1.439 (3)	C6—H6B	0.9700
O5—C9	1.452 (2)	C6—C7	1.506 (3)
O4—C7	1.213 (3)	C14—H14A	0.9700
O2—C3	1.208 (2)	C14—H14B	0.9700
O6—C11	1.209 (3)	C14—C13	1.551 (3)
O7—C11	1.333 (3)	C1—H1	0.9800
O7—C12	1.469 (2)	C19—H19	0.9300
O8—C21	1.360 (3)	C19—C18	1.425 (3)
O8—C20	1.366 (4)	C19—C20	1.322 (4)
C3—C2	1.495 (3)	C24—H24A	0.9600
C17—C5	1.565 (2)	C24—H24B	0.9600
C17—C16	1.566 (2)	C24—H24C	0.9600
C17—C1	1.549 (3)	C9—C13	1.522 (3)
C17—C24	1.544 (2)	C13—C12	1.546 (3)
C10—H10	0.9800	C23—H23A	0.9600
C10—C11	1.474 (3)	C23—H23B	0.9600
C10—C9	1.473 (3)	C23—H23C	0.9600
C2—H2A	0.9700	C23—C4	1.526 (3)
C2—H2B	0.9700	C4—C22	1.525 (3)
C2—C1	1.530 (3)	C25—H25A	0.9600
C5—H5	0.9800	C25—H25B	0.9600
C5—C6	1.555 (3)	C25—H25C	0.9600
C5—C4	1.558 (3)	C18—C21	1.346 (3)
C15—H15A	0.9700	C18—C12	1.496 (3)
C15—H15B	0.9700	C21—H21	0.9300
C15—C16	1.534 (3)	C22—H22A	0.9600
C15—C14	1.530 (3)	C22—H22B	0.9600
C26—H26A	0.9600	C22—H22C	0.9600
C26—H26B	0.9600	C20—H20	0.9300
C26—H26C	0.9600	C12—H12	0.9800
C26—C13	1.537 (3)	O9—H9A	0.854 (10)
C16—H16	0.9800	O9—H9B	0.87 (5)
C16—C8	1.567 (2)		
			
C3—O1—C4	127.99 (15)	O3—C1—C17	110.60 (15)
C1—O3—H3	109.5	O3—C1—C2	107.78 (16)
C10—O5—C9	61.24 (12)	O3—C1—H1	107.1
C11—O7—C12	120.34 (15)	C17—C1—H1	107.1
C21—O8—C20	105.6 (2)	C2—C1—C17	116.82 (16)
O1—C3—C2	121.08 (18)	C2—C1—H1	107.1
O2—C3—O1	116.68 (18)	O4—C7—C8	123.90 (18)
O2—C3—C2	122.2 (2)	O4—C7—C6	121.00 (19)
C5—C17—C16	105.25 (14)	C6—C7—C8	115.01 (17)
C1—C17—C5	110.46 (15)	C18—C19—H19	126.3
C1—C17—C16	106.34 (14)	C20—C19—H19	126.3
C24—C17—C5	116.71 (15)	C20—C19—C18	107.3 (3)
C24—C17—C16	111.46 (15)	C17—C24—H24A	109.5
C24—C17—C1	106.22 (15)	C17—C24—H24B	109.5
O5—C10—H10	116.6	C17—C24—H24C	109.5
O5—C10—C11	115.90 (18)	H24A—C24—H24B	109.5
O5—C10—C9	59.84 (12)	H24A—C24—H24C	109.5
C11—C10—H10	116.6	H24B—C24—H24C	109.5
C9—C10—H10	116.6	O5—C9—C10	58.92 (12)
C9—C10—C11	119.22 (18)	O5—C9—C8	114.78 (16)
C3—C2—H2A	108.6	O5—C9—C13	111.70 (15)
C3—C2—H2B	108.6	C10—C9—C8	119.47 (17)
C3—C2—C1	114.70 (17)	C10—C9—C13	116.83 (16)
H2A—C2—H2B	107.6	C13—C9—C8	119.51 (15)
C1—C2—H2A	108.6	C26—C13—C14	113.12 (16)
C1—C2—H2B	108.6	C26—C13—C12	109.14 (16)
C17—C5—H5	104.9	C9—C13—C26	110.22 (16)
C6—C5—C17	111.32 (15)	C9—C13—C14	108.71 (15)
C6—C5—H5	104.9	C9—C13—C12	107.95 (15)
C6—C5—C4	109.59 (14)	C12—C13—C14	107.54 (15)
C4—C5—C17	119.92 (15)	H23A—C23—H23B	109.5
C4—C5—H5	104.9	H23A—C23—H23C	109.5
H15A—C15—H15B	108.3	H23B—C23—H23C	109.5
C16—C15—H15A	109.9	C4—C23—H23A	109.5
C16—C15—H15B	109.9	C4—C23—H23B	109.5
C14—C15—H15A	109.9	C4—C23—H23C	109.5
C14—C15—H15B	109.9	O1—C4—C5	108.81 (14)
C14—C15—C16	109.15 (16)	O1—C4—C23	98.80 (15)
H26A—C26—H26B	109.5	O1—C4—C22	110.13 (16)
H26A—C26—H26C	109.5	C23—C4—C5	111.49 (17)
H26B—C26—H26C	109.5	C22—C4—C5	117.79 (16)
C13—C26—H26A	109.5	C22—C4—C23	108.17 (17)
C13—C26—H26B	109.5	C8—C25—H25A	109.5
C13—C26—H26C	109.5	C8—C25—H25B	109.5
O6—C11—O7	118.7 (2)	C8—C25—H25C	109.5
O6—C11—C10	122.6 (2)	H25A—C25—H25B	109.5
O7—C11—C10	118.69 (18)	H25A—C25—H25C	109.5
C17—C16—H16	104.2	H25B—C25—H25C	109.5
C17—C16—C8	116.73 (14)	C19—C18—C12	128.6 (2)
C15—C16—C17	118.95 (15)	C21—C18—C19	105.2 (2)
C15—C16—H16	104.2	C21—C18—C12	126.2 (2)
C15—C16—C8	106.81 (15)	O8—C21—H21	124.4
C8—C16—H16	104.2	C18—C21—O8	111.1 (2)
C7—C8—C16	103.57 (15)	C18—C21—H21	124.4
C7—C8—C9	112.87 (17)	C4—C22—H22A	109.5
C7—C8—C25	108.27 (16)	C4—C22—H22B	109.5
C9—C8—C16	107.88 (15)	C4—C22—H22C	109.5
C9—C8—C25	108.22 (16)	H22A—C22—H22B	109.5
C25—C8—C16	116.11 (16)	H22A—C22—H22C	109.5
C5—C6—H6A	109.0	H22B—C22—H22C	109.5
C5—C6—H6B	109.0	O8—C20—H20	124.6
H6A—C6—H6B	107.8	C19—C20—O8	110.8 (2)
C7—C6—C5	112.98 (16)	C19—C20—H20	124.6
C7—C6—H6A	109.0	O7—C12—C13	112.14 (15)
C7—C6—H6B	109.0	O7—C12—C18	104.05 (15)
C15—C14—H14A	108.7	O7—C12—H12	108.4
C15—C14—H14B	108.7	C13—C12—H12	108.4
C15—C14—C13	114.03 (16)	C18—C12—C13	115.24 (16)
H14A—C14—H14B	107.6	C18—C12—H12	108.4
C13—C14—H14A	108.7	H9A—O9—H9B	102 (4)
C13—C14—H14B	108.7		
			
O1—C3—C2—C1	62.1 (2)	C16—C8—C9—C10	−175.00 (16)
O5—C10—C11—O6	136.4 (2)	C16—C8—C9—C13	−18.7 (2)
O5—C10—C11—O7	−44.6 (2)	C8—C9—C13—C26	−70.9 (2)
O5—C10—C9—C8	−102.8 (2)	C8—C9—C13—C14	53.6 (2)
O5—C10—C9—C13	100.31 (18)	C8—C9—C13—C12	169.94 (17)
O5—C9—C13—C26	151.02 (16)	C6—C5—C4—O1	−152.42 (15)
O5—C9—C13—C14	−84.47 (18)	C6—C5—C4—C23	−44.5 (2)
O5—C9—C13—C12	31.9 (2)	C6—C5—C4—C22	81.4 (2)
O2—C3—C2—C1	−118.3 (2)	C14—C15—C16—C17	−150.82 (16)
C3—O1—C4—C5	−61.7 (2)	C14—C15—C16—C8	74.43 (19)
C3—O1—C4—C23	−178.13 (18)	C14—C13—C12—O7	172.34 (15)
C3—O1—C4—C22	68.7 (2)	C14—C13—C12—C18	−68.9 (2)
C3—C2—C1—O3	46.6 (2)	C1—C17—C5—C6	167.17 (16)
C3—C2—C1—C17	−78.6 (2)	C1—C17—C5—C4	−63.0 (2)
C17—C5—C6—C7	−53.6 (2)	C1—C17—C16—C15	51.7 (2)
C17—C5—C4—O1	77.03 (19)	C1—C17—C16—C8	−177.88 (15)
C17—C5—C4—C23	−175.05 (15)	C7—C8—C9—O5	−128.10 (17)
C17—C5—C4—C22	−49.2 (2)	C7—C8—C9—C10	−61.2 (2)
C17—C16—C8—C7	60.5 (2)	C7—C8—C9—C13	95.1 (2)
C17—C16—C8—C9	−179.60 (15)	C19—C18—C21—O8	−0.2 (3)
C17—C16—C8—C25	−58.0 (2)	C19—C18—C12—O7	38.5 (3)
C10—O5—C9—C8	110.76 (19)	C19—C18—C12—C13	−84.7 (3)
C10—O5—C9—C13	−109.10 (18)	C24—C17—C5—C6	−71.4 (2)
C10—C9—C13—C26	85.9 (2)	C24—C17—C5—C4	58.4 (2)
C10—C9—C13—C14	−149.55 (17)	C24—C17—C16—C15	−63.7 (2)
C10—C9—C13—C12	−33.2 (2)	C24—C17—C16—C8	66.8 (2)
C5—C17—C16—C15	168.90 (15)	C24—C17—C1—O3	168.87 (15)
C5—C17—C16—C8	−60.7 (2)	C24—C17—C1—C2	−67.4 (2)
C5—C17—C1—O3	−63.67 (19)	C9—O5—C10—C11	110.20 (19)
C5—C17—C1—C2	60.1 (2)	C9—C10—C11—O6	−155.2 (2)
C5—C6—C7—O4	−120.2 (2)	C9—C10—C11—O7	23.8 (3)
C5—C6—C7—C8	56.6 (2)	C9—C8—C7—O4	4.7 (3)
C15—C16—C8—C7	−163.55 (15)	C9—C8—C7—C6	−171.99 (16)
C15—C16—C8—C9	−43.7 (2)	C9—C13—C12—O7	55.2 (2)
C15—C16—C8—C25	77.9 (2)	C9—C13—C12—C18	173.99 (17)
C15—C14—C13—C26	100.8 (2)	C4—O1—C3—O2	−176.60 (18)
C15—C14—C13—C9	−22.0 (2)	C4—O1—C3—C2	3.0 (3)
C15—C14—C13—C12	−138.65 (16)	C4—C5—C6—C7	171.35 (17)
C26—C13—C12—O7	−64.6 (2)	C25—C8—C7—O4	−115.1 (2)
C26—C13—C12—C18	54.2 (2)	C25—C8—C7—C6	68.2 (2)
C11—O7—C12—C13	−40.8 (2)	C25—C8—C9—O5	−8.3 (2)
C11—O7—C12—C18	−166.02 (17)	C25—C8—C9—C10	58.6 (2)
C11—C10—C9—O5	−104.7 (2)	C25—C8—C9—C13	−145.11 (17)
C11—C10—C9—C8	152.49 (18)	C18—C19—C20—O8	−1.1 (3)
C11—C10—C9—C13	−4.4 (3)	C21—O8—C20—C19	1.0 (3)
C16—C17—C5—C6	52.78 (18)	C21—C18—C12—O7	−141.6 (2)
C16—C17—C5—C4	−177.44 (15)	C21—C18—C12—C13	95.2 (3)
C16—C17—C1—O3	50.0 (2)	C20—O8—C21—C18	−0.5 (3)
C16—C17—C1—C2	173.79 (15)	C20—C19—C18—C21	0.8 (3)
C16—C15—C14—C13	−38.0 (2)	C20—C19—C18—C12	−179.3 (2)
C16—C8—C7—O4	121.1 (2)	C12—O7—C11—O6	179.16 (18)
C16—C8—C7—C6	−55.6 (2)	C12—O7—C11—C10	0.1 (3)
C16—C8—C9—O5	118.09 (16)	C12—C18—C21—O8	179.87 (19)

### Hydrogen-bond geometry (Å, °)

**Table:**

*D*—H···*A*	*D*—H	H···*A*	*D*···*A*	*D*—H···*A*
O3—H3···O9	0.82	2.04	2.817 (3)	158
O9—H9A···O6^i^	0.85 (1)	2.00 (1)	2.841 (3)	169 (4)
O9—H9B···O4^ii^	0.87 (5)	2.33 (5)	3.124 (3)	152 (4)

Symmetry codes: (i) −*x*+1/2, −*y*+1, *z*−1/2; (ii) *x*+1/2, −*y*+3/2, −*z*+1.
